# Sarcopenia and cognitive performance in hospitalized older adults: evidence of an association

**DOI:** 10.3389/frdem.2025.1713418

**Published:** 2026-01-08

**Authors:** Otávio Augusto Garcia Simili, Lorena Natalino Haber Garcia, Caroline Sollis, Sandra Maria Barbalho, Marcelo Dib Bechara, Caroline Barbalho Lamas, Claudia Rucco Penteado Detregiachi, Karina Quesada

**Affiliations:** 1 Department of Biochemistry and Pharmacology, School of Medicine and Nutrition, University of Marília (UNIMAR), Marília, São Paulo, Brazil; 2 Postgraduate Program in Structural and Functional Interactions in Rehabilitation, School of Medicine, University of Marília (UNIMAR), Marília, São Paulo, Brazil; 3 Department of Gerontology, School of Gerontology, Universidade Federal de São Carlos (UFSCar), São Carlos, São Paulo, Brazil

**Keywords:** sarcopenia, cognitive function, hospitalized older adults, mini-mental state examination, muscle strength

## Abstract

**Introduction:**

Population aging increases the coexistence of geriatric syndromes, including sarcopenia and cognitive impairment, which negatively affect functional prognosis and clinical outcomes. Evidence on the association between sarcopenia and cognitive performance in hospitalized elderly individuals, particularly across cognitive domains, remains limited. Therefore, the objective of this study was to investigate the association between sarcopenia and cognitive performance, both globally and across specific cognitive domains, in hospitalized elderly individuals.

**Methods:**

This observational cross-sectional study included elderly patients hospitalized in a medium-sized hospital in São Paulo, Brazil, from 2024 to 2025, evaluated within the first 48 hours of admission. Exclusion criteria included weight-loss diets, chronic renal failure on dialysis, paresis or hemiparesis due to stroke, altered body fluid levels, pacemaker use, and chronic corticosteroid therapy. Sarcopenia screening and diagnosis followed the European Consensus criteria, and cognitive performance was assessed using the Mini-Mental State Examination (MMSE). Comparative analyses were conducted among non-sarcopenic, sarcopenia risk, and sarcopenic groups, including correlations with sociodemographic variables.

**Results:**

Eighty-four older adults were assessed (54.76% women; mean age 68.1 ± 6.7 years). Sarcopenia risk was identified in 29.76% and confirmed sarcopenia in 19.05%. MMSE scores showed a moderate negative correlation with age (*r* = −0.48; *p* < 0.0001) and positive correlations with education (*r* = 0.58; *p* < 0.0001), physical activity (*r* = 0.31; *p* = 0.0038), and income (*r* = 0.23; *p* = 0.0326). Mean MMSE scores differed significantly among groups: non-sarcopenic (26.84 ± 3.24), sarcopenia risk (22.32 ± 5.86), and sarcopenic (18.69 ± 7.98) (*p* < 0.0001). Worse performance in orientation, attention/calculation, and language was observed in the sarcopenia risk and sarcopenia groups (*p* < 0.001).

**Conclusion:**

Sarcopenia, even at early stages, is associated with reduced global cognitive performance and specific impairments in orientation, attention/calculation, and language in hospitalized elderly individuals. These findings underscore the importance of early screening and targeted interventions to preserve both muscle and cognitive health, reinforcing the need for integrated care strategies in hospital settings.

## 1 Introduction

Population aging is a global phenomenon that poses significant challenges to health systems, particularly in maintaining the functionality, autonomy, and quality of life of older adults (Corrao et al., 2025). In this context, sarcopenia, characterized by the progressive and widespread loss of muscle mass, strength, and function, has emerged as a major public health challenge (Cruz-Jentoft et al., 2019).

The prevalence of sarcopenia increases proportionally with age and is associated with a series of adverse outcomes, including functional decline, increased risk of falls and fractures, loss of independence, and increased mortality (Kirk et al., 2024). Global estimates indicate a prevalence ranging from 2 to 17%, with an average of approximately 10% (Petermann-Rocha et al., 2021). In Brazil, this rate is even higher, reaching 28.6% in certain studies, depending on the diagnostic criteria used (Diz et al., 2016), which highlights the urgency of adequate diagnostic and preventive strategies.

Aging is a significant and independent cause for the development of sarcopenia, contributing to marked functional limitations and worsening of pre-existing clinical conditions, especially in hospitalized elderly individuals (Fernandes et al., 2022; Veronese et al., 2019). Furthermore, it is associated with unfavorable clinical outcomes, including longer hospital stays, an increased risk of complications, and higher healthcare costs (Álvarez-Bustos et al., 2022; Tan et al., 2017). The severity of this condition justifies recent efforts to standardize its definition and diagnosis.

In recent decades, advances in the field have culminated in the development of international consensus guidelines that standardize the definition and diagnosis of sarcopenia. The 2019 European Consensus, for example, introduced a staged classification (probable, confirmed, and severe), facilitating clinical practice and therapeutic targeting (Cruz-Jentoft et al., 2019). The 2024 International Consensus reaffirms the need to simultaneously consider loss of muscle mass, strength, and function in the diagnosis (Kirk et al., 2024).

In addition to its physical impacts, sarcopenia has been increasingly associated with cognitive impairment, including dementia, neuropsychiatric disorders, and brain atrophy (Huang et al., 2025; Tolea and Galvin, 2015). This bidirectional relationship between muscle function and cognitive health underscores the importance of integrated assessments. To measure cognitive performance, the Mini-Mental State Examination (MMSE) stands out as a validated instrument that assesses domains such as orientation, visuospatial constructive ability, immediate memory, recall, attention, and calculation, in addition to language (Wu et al., 2024).

However, although the literature recognizes the correlation between sarcopenia and cognitive decline, the most affected domains in hospitalized elderly individuals with sarcopenia have not yet been clarified. Determining which areas of cognitive functioning are most impacted can inform personalized clinical interventions and strengthen interdisciplinary approaches in the hospital setting.

Therefore, the objective of this study was to investigate the association between sarcopenia and cognitive performance, both globally and by domains, in hospitalized elderly individuals.

## 2 Materials and methods

### 2.1 Study design

This observational, cross-sectional study was conducted in a medium-sized hospital located in the interior of the state of São Paulo, Brazil, between September 2024 and May 2025.

### 2.2 Participants

Patients aged 60 years or older, of both sexes, hospitalized in clinical wards and evaluated within the first 48 h of admission were included. Inclusion criteria required sufficient clinical and cognitive ability to understand and actively participate in the administration of the instruments. Participation was voluntary and subject to signing the Informed Consent Form (ICF). Patients undergoing palliative care; with severe physical limitations that prevented assessment of muscle strength or cognitive status; with pacemakers or metal valves; with generalized edema or anasarca; undergoing renal replacement therapy; on severely restricted diets; on prolonged use of corticosteroids; with paresis or plegia; inability to remain upright; neuromuscular diseases; suspected or confirmed highly transmissible infectious diseases or in isolation; and those with advanced dementia syndromes or inability to respond for themselves were excluded.

The study included 105 elderly patients admitted to a medium-sized hospital in the interior of São Paulo state, Brazil. Of these, 84 met the eligibility criteria and underwent the proposed assessments. Those hospitalized for up to 48 h were considered eligible to minimize biases related to prolonged hospitalization.

### 2.3 Data collection procedures

The sample selection was based on screening of electronic hospital records. After identifying eligible patients, the researchers employed a face-to-face bedside approach to present the research objectives. Those who understood the proposal and consented to participate signed the informed consent form.

Data collection involved structured interviews to obtain sociodemographic information (age, sex, education, origin), clinical information (comorbidities, continuous use of medications, history of hospitalizations and falls), and behavioral information (smoking, alcohol consumption and physical activity).

#### 2.3.1 Assessment of sarcopenia

Sarcopenia was investigated according to the guidelines of the European Working Group on Sarcopenia in Older People 2 (Cruz-Jentoft et al., 2019), through a three-stage protocol: Stage 1 consisted of screening using the SARC-F questionnaire (Strength, Assistance with walking, Rise from a chair, Climb stairs, and Falls), consisting of five items on strength, assistance with locomotion, ability to get up from a chair, climb stairs, and occurrence of falls in the last 12 months. The score ranges from 0 to 10, with a score of ≥ 4 indicating a risk of sarcopenia. Stage 2 involved assessing muscle strength/dynamometry, with handgrip strength measured using a hand dynamometer (Jamar) according to a standardized protocol. Three alternating measurements were taken on each hand, with a minimum interval of 1 min to avoid fatigue. Values were expressed in kilogram-force (kgf) and calculated as the average of three measurements of the dominant hand.

The adopted cutoff points were <27 kgf for men and <16 kgf for women. In the third stage, to confirm sarcopenia by detecting low muscle quantity/quality, a tetrapolar bioelectrical impedance device (Biodynamics®, model 450) was used. Individuals with muscle mass <20 kg (men) and <15 kg (women) were considered sarcopenic, according to the equation by Janssen et al. (2000).

#### 2.3.2 Assessment of cognition

Cognitive function was assessed using the Mini-Mental State Examination (MMSE), which comprises tests of orientation (temporal and spatial), immediate and delayed memory, attention, calculation, language, comprehension, and motor skills. The maximum score is 30 points, and the following categories were adopted to classify cognitive performance: ≥ 25 points (preserved cognition), 21–24 (mild impairment), 10–20 (moderate impairment), and < 9 points (severe impairment).

In the moment of MMSE application, the data about muscle mass, strength and sarcopenia status were not available yet to the test performers. The results were appreciated only once the MMSE was concluded.

### 2.4 Data processing and statistical analysis

After collection, the data were anonymized and tabulated in a spreadsheet (Microsoft Excel®). Statistical analysis was conducted using BioEstat® software, version 5.3. Comparison tests between groups and correlation analysis were applied according to data distribution, with a significance level set at *p* < 0.05. The following statistical tests were used: Spearman’s correlation test and Mann–Whitney test.

### 2.5 Ethical aspects

The study was approved by the Research Ethics Committee of the University of Marília, São Paulo, Brazil, under opinion number 5.467.081 em 13/06/2022.

## 3 Results

The paragraph was included in the Methods section, 2.2 Participants.

Initial screening for sarcopenia was performed using the SARC-F questionnaire, with a mean score of 2.46 ± 2.68. Twenty-five individuals (29.76%) obtained a score ≥ 4 and were classified as at risk for sarcopenia. Handgrip strength allowed for stratification of the sample into two groups: those with and those without dynapenia. Forty-two elderly individuals (50%) were identified with reduced muscle strength, including 24 women (57.14%) and 18 men (42.86%). Body composition analysis, obtained by bioelectrical impedance analysis, identified 16 participants (19.05%) with reduced appendicular skeletal muscle mass. Based on handgrip strength and muscle mass parameters, the sample was classified into three groups: healthy (*n* = 37), at risk of sarcopenia (*n* = 31), and sarcopenic (*n* = 16).

Table 1 presents the sociodemographic characteristics of the sample. There was a predominance of females (54.76%), an age range from 60 to 69 years (51.19%), and self-reported white skin color (67.86%). Regarding education, more than half had completed elementary school at most, and 21.43% were illiterate. Most participants reported a monthly income of less than two minimum wages (79.77%) and a lack of regular physical activity (60.71%).

**Table 1:** Sociodemographic characterization of hospitalized elderly individuals participating in the study (*n* = 84).

Variables	Subgroups	Absolute frequency (*n*)	Relative frequency (%)	Total
Sex	Male	38	45.24%	100%
	Female	46	54.76%	
Age	60–69 years	43	51.19%	100%
	70–79 years	29	34.52%	
	≥ 80 years	12	14.29%	
Ethnicity/skin color	White	57	67.86%	100%
	Black	6	7.14%	
	Brown/mixed-race	19	22.62%	
	Yellow	1	1.19%	
	Not Declared	1	1.19%	
Education	Illiterate	18	21.43%	100%
	Primary school (1st stage)	25	29.76%	
	Primary school (2nd stage)	10	11.90%	
	High school	20	23.81%	
	College	11	13.10%	
	Postgraduate	0	0%	
Income	< 1 minimum wage	35	41.67%	100%
	>1 e ≤ 2 minimum wage	32	38.10%	
	>2 e ≤ 3 minimum wage	8	9.52%	
	> 3 minimum wage	8	9.52%	
	Not reported	1	1.19%	
Physical activity	No	51	60.71%	100%
	Yes	33	39.29%	
	Daily	12	14.29%	
	3-5×/week	12	14.29%	
	1-2×/week	9	10.71%	
Smoking status	Non-smoker	38	45.24%	100%
	Smoker	9	10.71%	
	Former smoker	37	44.05%	
Alcohol consumption	Yes	28	33.33%	100%
	No	56	66.67%	

The Mini-Mental State Examination (MMSE) was administered to the three groups defined by muscle status. Table 2 presents the correlation coefficients between cognitive scores and sociodemographic variables. A moderate negative correlation was observed between age and cognitive performance (*r* = −0.4799, *p* < 0.0001), indicating a progressive decline in cognitive performance associated with aging. Education demonstrated a significant positive correlation (*r* = 0.5842; *p* < 0.0001), suggesting a protective effect of education on cognitive domains. Positive correlations, although of lesser magnitude, were also observed with physical activity (*r* = 0.3124; *p* = 0.0038), alcohol consumption (*r* = 0.3235; *p* = 0.0027) and monthly income (*r* = 0.2333; *p* = 0.0326). No significant correlations were observed for the variables sex (*r* = 0.0496; *p* = 0.6542) and smoking (*r* = −0.0154; *p* = 0.8896).

**Table 2:** Correlation between the score obtained in the mini-mental assessment test and the sociodemographic variables evaluated.

Variables	Coefficient (*r*)	*p*-value
Sex	0.0496**	0.6542
Age	−0.4799**	< 0.0001
Education	0.5842**	< 0.0001
Income	0.2333**	0.0326
Physical activity	0.3124**	0.0038
Smoking status	−0.0154**	0.8896
Alcohol consumption	0.3235**	0.0027

^**^Spearman’s rank correlation test.

The analysis of the MMSE means between sociodemographic groups, presented in Table 3, revealed statistically significant differences according to age, education, income, physical activity, and alcohol consumption. Individuals aged 60 to 69 years had a higher mean score (25.74 ± 4.47) than those aged ≥ 70 years (21.39 ± 6.98; *p* = 0.0010). Cognitive performance increased progressively with education level, with participants with elementary school II standing out (27.05 ± 2.98), while illiterate individuals had the lowest scores (20.35 ± 6.70; *p* < 0.0001). Elderly individuals with an income higher than two minimum wages demonstrated higher cognitive performance (27.44 ± 2.42) compared to those with lower incomes (22.72 ± 6.48; *p* = 0.0034). Regular physical activity was associated with higher scores (26.15 ± 3.77) compared to inactive individuals (21.98 ± 6.90; *p* = 0.0047).

**Table 3:** Comparison of the mean score on the mini-mental state examination according to sociodemographic variables (*n* = 84).

Variables	Subgroups	Mean ± Standard deviation (Median)	*p*-value
Sex	Male	24.105 ± 6.115 (26)	0.6532
	Female	23.217 ± 6.300 (26)	
Age	60–69 years	25.744 ± 4.467 (27)	0.0010
	≥ 70 years	21.390 ± 6.982 (22)	
Education	Illiterate and primary school (1st stage)	20.349 ± 6.704 (21)	< 0.0001
	Primary school (2nd stage), high school, college e postgraduate studies	27.049 ± 2.983 (28)	
Income	≤2 minimum wages	22.721 ± 6.476 (25)	0.0034
	>2 minimum wages	27.438 ± 2.421 (28.5)	
Physical activity	No	21.980 ± 6.904 (24)	0.0047
	Yes	26.152 ± 3.768 (27)	
	Daily		
	3–5×/week		
	1–2×/week		
Smoking status	Non-smoker	23.474 ± 7.005 (26)	0.8892
	Smoker or former smoker	23.739 ± 5.515 (26)	
Alcohol consumption	Yes	26.357 ± 3.803 (28)	0.0034
	No	21 ± 6.716 (24.5)	

^**^Mann–Whitney *U*-test.

The mean score on the Mini-Mental State Examination (MMSE) varied significantly between the groups defined by the risk or presence of sarcopenia, as shown in Table 4. Non-sarcopenic individuals had a higher mean score (26.84 ± 3.24), followed by those at risk (22.32 ± 5.86) and sarcopenic individuals (18.69 ± 7.98). There was a statistically significant difference between non-sarcopenic and at-risk individuals (*p* = 0.0003), as well as between non-sarcopenic and sarcopenic individuals (*p* < 0.0001). No significant difference was observed between the at-risk and sarcopenic groups (*p* = 0.1269). When considered together, at-risk or sarcopenic individuals obtained a significantly lower score (21.09 ± 6.80) compared to non-sarcopenic individuals (26.84 ± 3.24; *p* < 0.0001).

**Table 4:** Comparison of mini-mental state examination scores in non-sarcopenic elderly individuals at risk of sarcopenia and sarcopenic individuals (*n* = 84).

Variables	Non-saropênicos (*n* = 37)	Risk of sarcopenia (*n* = 31)	Sarcopenic (*n* = 16)	*p*-value
	Mean ± standard deviation (Median)			
MMSE score	26.838 ± 3.236 (28)	22.323 ± 5.856 (24)	18.688 ± 7.981 (18.5)	0.0003*^1^
				< 0.0001*^2^
				0.1269*^3^

*Mann–Whitney *U*-test.

1 Comparison between non-sarcopenic and at-risk-of-sarcopenia groups.

2 Comparison between non-sarcopenic and sarcopenic groups.

3 Comparison between at-risk of sarcopenia and sarcopenic groups.

**Figure 1:**
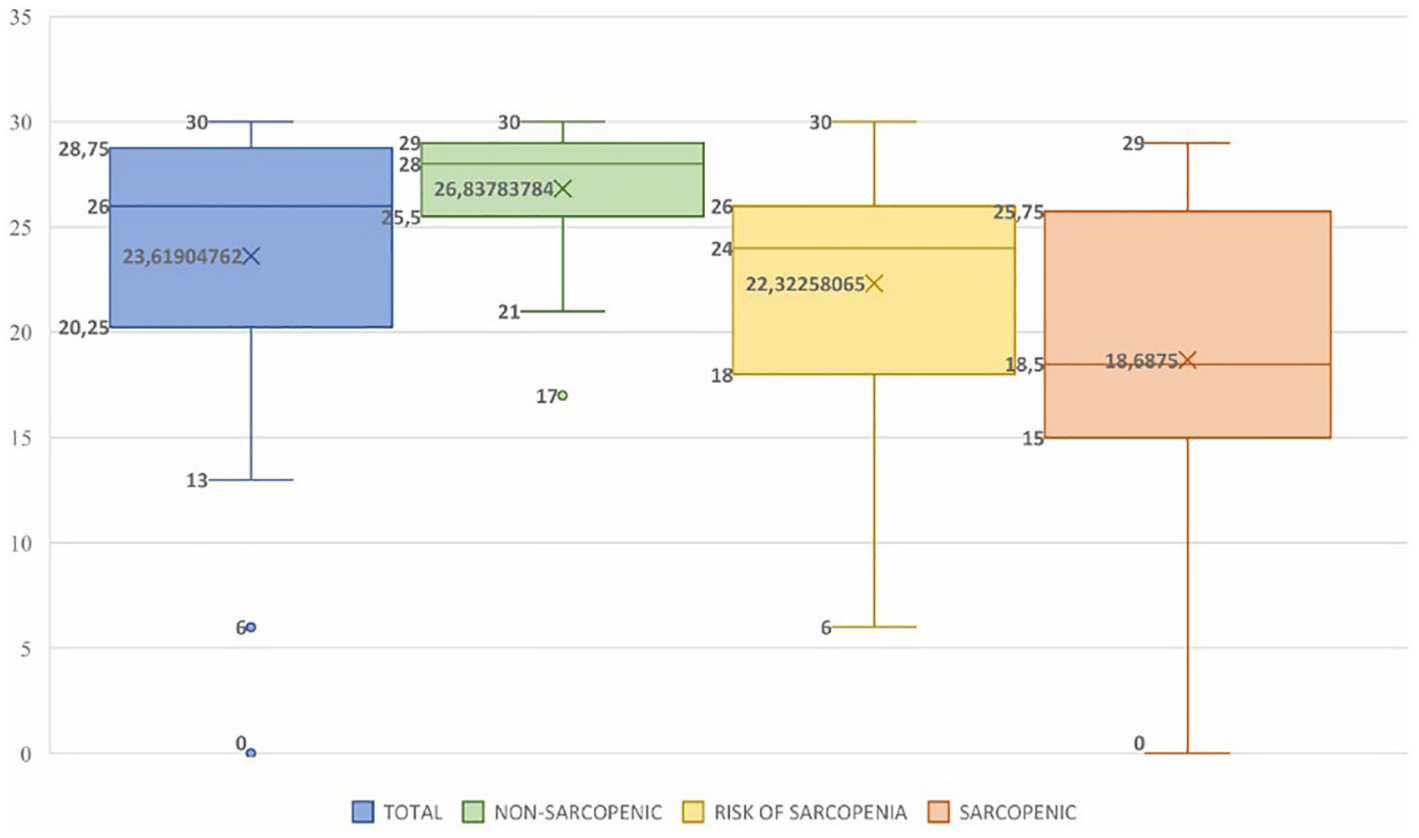
Distribution of Mini-Mental State Examination (MMSE) scores according to sarcopenic classification in hospitalized elderly individuals. Boxplot representing the distribution of MMSE scores in non-sarcopenic at-risk and sarcopenic elderly individuals. The center line of each box indicates the median. The upper and lower limits represent the 1st and 3rd quartiles, and the ends of the bars indicate the minimum and maximum values within 1.5 times the interquartile range. Points outside the bars represent outliers. A trend toward progressive reduction in cognitive scores is observed as the sarcopenic state worsens, with the lowest values identified in the sarcopenic group.

Figure 1 illustrates the distribution of MMSE scores across groups. A trend toward progressive decline in cognitive performance was observed as muscle condition worsened. The healthy group had the highest median (26.8 points), followed by the at-risk group (22.3 points), while the sarcopenic group had the lowest (18.6 points). Furthermore, the interquartile range was wider in the at-risk and sarcopenic groups, reflecting greater cognitive variability.

In the analysis of the MMSE domains, patients without signs of sarcopenia were compared to those classified as at risk or sarcopenic. The domains evaluated were: temporal–spatial orientation, registration, attention and calculation, remote memory, and language. Significant differences were observed for orientation, attention, calculation, and language (*p* = 0.0003, *p* = 0.0015, and *p* < 0.0001, respectively). The registration (*p* = 0.4778) and remote memory (*p* = 0.0752) domains did not present statistically significant differences (Table 5).

**Table 5:** Comparison of the score obtained in each domain assessed in the mini-mental test between non-sarcopenic elderly people and those at risk or with sarcopenia.

Variables	Non-sarcopenic (*n* = 37)	Risk of sarcopenia and sarcopenia (*n* = 47)	*p*-value
MMSE domains	Mean ± Standard deviation		
Orientation	9.595 ± 0.686	7.468 ± 2.896	0.0003**
Registration	2.892 ± 0.393	2.766 ± 0.598	0.4878**
Atention/calculation	4.054 ± 1.632	2.362 ± 2.317	0.0015**
Remote memory	1.703 ± 1.024	1.277 ± 1.036	0.0752**
Language	8.595 ± 0.686	7.213 ± 1.887	<0.0001**

Mann–Whitney test.

## 4 Discussion

This study revealed a high prevalence of sarcopenia and dynapenia in elderly hospitalized patients, demonstrating a significant association between muscle status and cognitive decline, particularly in the domains of attention/calculation, orientation, and language. Our findings suggest that sarcopenia is not only associated with loss of muscle mass and function but may also negatively impact specific cognitive functions, reinforcing the importance of assessing sarcopenia in hospital settings.

Considering the accelerated aging of the population and the consequent increase in the prevalence of chronic conditions, it is urgent to understand how the loss of muscle mass and function can negatively impact cognition in clinical settings. Growing evidence indicates that sarcopenia is not limited to physical consequences but is also related to neurocognitive changes, suggesting a multifactorial interaction that compromises autonomy and quality of life in aging (Hsu et al., 2024; Kannan and Arunachalam, 2025; Kim et al., 2024).

Half of the individuals with sarcopenia had moderate cognitive impairment, and 12.5% had severe impairment, while only 31.25% maintained preserved cognition. In contrast, in the group without sarcopenia, 83.78% had preserved cognitive function, and no cases of severe impairment were identified. These results reinforce the hypothesis that the severity of sarcopenia is proportionally associated with the magnitude of cognitive decline.

In the sarcopenia risk group, cognitive changes were also significant, with only 45.16% of individuals showing preserved cognition, and a significant distribution between mild (22.58%), moderate (20.32%), and severe (22.58%) levels of impairment. These data reinforce the hypothesis that even the risk of sarcopenia is already associated with cognitive impairments, possibly related to standard pathophysiological mechanisms, such as low-grade chronic inflammation, oxidative stress, metabolic dysfunction, and reduced physical activity (Arosio et al., 2023; Yang et al., 2022; Shlisky et al., 2017).

Similar results were observed by Li et al. (2022), who identified a significant association between sarcopenia and cognitive decline, particularly in the domains of attention and calculation, in hospitalized elderly Chinese individuals. Similarly, Dong et al. (2024) demonstrated that individuals with sarcopenia had lower scores in executive functions, verbal fluency, calculation, and delayed recall memory, reinforcing the hypothesis that muscle loss influences multiple cognitive domains.

Physical activity showed a moderate correlation with Mini-Mental State Examination scores (*r* = 0.3124), suggesting a positive association between higher activity levels and better cognitive performance. It is noteworthy that a sedentary lifestyle was prevalent in more than half of the sample (60.71%), reinforcing the vulnerability of this population to neurocognitive impairment. The literature has consistently highlighted the importance of regular physical exercise, especially when combined with a balanced diet, as a key protective factor for brain function in older adults. This beneficial effect is partially mediated by the release of neuroactive myokines, such as irisin, cathepsin B, BDNF (Brain-Derived Neurotrophic Factor), IL-6, and LIF, produced by skeletal muscle during muscle contraction. These substances promote the neuroplasticity of synaptic function and cognitive integrity throughout the aging process. Conversely, the reduction in muscle mass often observed in physically inactive individuals compromises the release of these myokines and may contribute to the acceleration of cognitive decline (Arosio et al., 2023; Scisciola et al., 2021; Sui et al., 2020; Lim et al., 2022).

Recent evidence indicates that higher educational attainment is positively associated with gray matter volume in several cortical regions, which appears to mediate the prevention of sarcopenia in older adults indirectly. This relationship suggests a bidirectional communication between brain and muscle, in which the central nervous system regulates muscle function through neuromuscular junctions, while muscle tissue influences brain function through the release of cytokines such as myokines (Arosio et al., 2023; Zhang et al., 2024; Lu et al., 2024). In the present study, it was observed that more than 50% of the participants had 4 years of schooling or less, which was strongly associated with the presence of sarcopenia. These data corroborate the findings of Gabriela et al. (2023), that who in an observational study conducted with elderly Brazilians, identified that 61.7% of individuals with sarcopenia had between one and 4 years of schooling. Thus, education, as a marker of cognitive capital and a socioeconomic indicator, should be considered a critical variable in the formulation of preventive strategies and public policies aimed at reducing sarcopenia in the elderly population.

Furthermore, higher health literacy is associated with better awareness and understanding of clinical conditions such as sarcopenia. Individuals with higher educational levels tend to adopt healthier lifestyles characterized by a balanced diet and regular physical activity—key factors in preventing and managing this condition (Rao et al., 2025; Wang et al., 2024).

A comparative analysis between the groups revealed statistically significant differences in Mini-Mental State Examination (MMSE) scores, with lower scores observed in individuals at risk for or with a confirmed diagnosis of sarcopenia compared to those without the condition (Table 4 and Figure 1). These results corroborate previous findings in the literature, which indicate a dose-dependent association between sarcopenia severity and cognitive impairment (Peng et al., 2019). Furthermore, data from studies suggest that individuals with sarcopenia have a two-fold increased risk of impaired cognitive function, with this risk significantly increased in cases of severe sarcopenia (Ohta et al., 2023).

In a longitudinal study conducted with a Chinese population (Zhang et al., 2023), demonstrated a bidirectional relationship between sarcopenia and cognitive function. Primary sarcopenia was associated with lower subsequent cognitive performance, while lower initial cognitive scores also predicted a higher risk of future sarcopenia, suggesting a feedback dynamic between both conditions. Furthermore, Yigit et al. (2022) observed that elderly individuals with cognitive impairment had a significantly higher prevalence of sarcopenia (15.4%) compared to those with preserved cognition (3.7%), reinforcing the existence of a clinically relevant association (Zhang et al., 2023; Yigit et al., 2022).

Analysis of the cognitive domains assessed by MMSE revealed statistically significant differences between the study groups. Individuals classified as “at risk for sarcopenia” and “sarcopenic” presented lower performance in the domains of orientation (*p* = 0.0003), attention/calculation (*p* = 0.0015), and language (*p* < 0.0001) compared to non-sarcopenic individuals. These results suggest an association between the presence of sarcopenia and cognitive impairment, reinforcing the hypothesis that muscle mass loss may be related to deficits in specific cognitive functions. This finding highlights the importance of cognitive assessments in elderly individuals with sarcopenia, although confounding factors and longitudinal studies are needed to confirm this association.

In the language domain, the difference observed in our study was statistically significant, confirming the results of the English Longitudinal Study of Ageing (ELSA), which demonstrated an association between sarcopenia and impairments in multiple cognitive domains, particularly verbal fluency. In this study, individuals with sarcopenia were more likely to perform poorly on verbal fluency tests, reinforcing the hypothesis that reduced muscle mass and strength can directly impact language skills, consequently affecting the autonomy of older adults (Maniscalco et al., 2024).

Our study has some limitations. First, it is a cross-sectional study that only observed patients up to 48 h after hospitalization, making it impossible to draw causal conclusions. Second, it was conducted with a sample from a single center. Finally, it uses only one screening instrument to assess the degree of global cognition (MMSE), which may limit the ability to detect specific interferences in the cognitive domains addressed. Overall, these limitations suggest the need for caution in interpreting the results and for further research in this area.

It is important to acknowledge the limitations in this present study. First, it must be clear about the limitation showed by the small sample selected for the statistical tests, specifically the small number of participants included in the sarcopenic subgroup. A further limitation concerns the exclusive use of the MMSE in a sample with widely varying educational levels. Because the MMSE does not incorporate education-adjusted cutoffs in its scoring, cognitive status may have been underestimated among participants with lower schooling, thereby limiting the interpretability of the results. Additionally, the domain-specific analyses should be interpreted with caution, as MMSE subdomains have limited psychometric validity for fine-grained cognitive profiling. Future studies should employ neurocognitive instruments that provide more robust adjustment for education and allow a more precise assessment of specific cognitive domains.

Another important limitation of this study is the absence of multivariable adjustments, which prevents conclusions about independent relationships between sarcopenia and cognitive performance. Although meaningful associations were identified, these findings may be partially influenced by unmeasured or uncontrolled confounders. Future studies with larger samples and sufficient statistical power are needed to apply multivariate models capable of clarifying the relative contributions of demographic, clinical, and functional factors to the associations observed.

It is important to acknowledge that bioelectrical impedance analysis (BIA) is sensitive to variations in hydration status, which may compromise the accuracy of body composition estimates in hospitalized individuals. Although the study adopted exclusion criteria related to evident hydration disturbances, subclinical fluctuations may still occur during hospitalization and influence parameters such as fat-free mass and total body water. This methodological limitation should be considered when interpreting the results. Future investigations may incorporate reference techniques (e.g., DEXA or computed tomography) to enhance measurement precision and reduce potential biases.

Overall, these findings underscore that sarcopenia, even in its early stages, is significantly associated with global cognitive impairment in hospitalized elderly individuals, with notable deficits in attention/calculation and language domains. This highlights the importance of early screening for sarcopenia in clinical settings, enabling timely interventions that may simultaneously preserve muscle function and cognitive health. Future studies employing longitudinal designs and comprehensive cognitive assessments are warranted to further elucidate these associations.

It is important to distinguish statistical significance from clinical relevance when interpreting the findings of this study. Although some differences between groups reached statistical significance, the magnitude of these effects must be evaluated in the context of their actual clinical impact on the population assessed. In hospitalized older adults, small variations in muscle or cognitive parameters may achieve statistical significance due to the sensitivity of the tests employed, yet may not represent changes substantial enough to modify clinical decision-making or prognosis. Therefore, the results should be understood as indicative of meaningful trends, while their clinical applicability must be interpreted with caution and complemented by future investigations capable of more robustly quantifying the clinical weight of these associations.

## Data Availability

The original contributions presented in the study are included in the article/supplementary material, further inquiries can be directed to the corresponding author.
